# 373. An open-label 24-week randomised multicentered clinical trial of Bictegravir/Emtricitabine/Tenofovir Alafenamide compared to Tenofivir/Lamifuvine/Efavirenz as an initial regimen in Chinese late presenters with HIV-1 infection

**DOI:** 10.1093/ofid/ofad500.443

**Published:** 2023-11-27

**Authors:** Ling Qin, Ruichao Lu, Rugang Wang, Yingquan Zhou, Hongxia Wei, Ping Ma, Junyan Zhang, Wei Lyu

**Affiliations:** Peking Union Medical College Hospital, Chinese Academy of Medical Science and Peking Union Medical College, Beijing, Beijing, China; Guangxi Zhuang Autonomous Region Longtan Hospital, Liuzhou, Guangxi, China; Dalian Public Health Clinical Center, Dalian, Liaoning, China; Lanzhou Pulmonary Hospital Lanzhou, Lanzhou, Gansu, China; The Second Hospital of Nanjing, Nanjing University of Chinese Medicine, Nanjing, Jiangsu, China; Nankai University Second People's Hospital, School of Medicine, Nankai University, tianjin, Tianjin, China; Shanxi Bethune Hospital, Shanxi Academy of Medical Sciences, shanghai, Shanghai, China; Peking Union Medical College Hospital, Chinese Academy of Medical Science and Peking Union Medical College, Beijing, Beijing, China

## Abstract

**Background:**

Despite tremendous efforts, late presenters (CD4 ≤ 350 cells/μl) still account for a staggering proportion of HIV patients at the time of diagnosis. The main objective of this study was to evaluate the viral-immunological efficacy of bictegravir/emtricitabine/ tenofovir alafenamide (BIC/FTC/TAF) in treatment-naïve, late presenters in Chinese patients with HIV-1 infection, comparing with national free tenofovir disoproxil fumarate/lamivudine/efavirenz/(TDF/3TC/EFV).

**Methods:**

We conducted an open-label, randomized controlled trial at six HIV care centers in China, starting recruitment from June 2021 to December 2022. All treatment-naïve late presenters were randomised to receive TDF/3TC/EFV (group A) or BIC/FTC/TAF (group B). Serum HIV loads and CD4 cell counts were measured at baseline, 4 weeks, 12 weeks, and 24 weeks. The proportion of HIV-RNA < 50 copies/ml at 12 weeks was considered the primary endpoint and changes in viral loads and CD4 cell counts at other time points were secondary outcomes in PP and ITT analysis.

**Results:**

We enrolled 200 late presenters, randomizing to group A (n=99) and group B (n=101). As of April 2023, 178/200 (89%) and 156/200 (78%) of randomized individuals completed 12 weeks and 24 weeks of follow-up. The study groups (n=200) were similar at baseline with a mean age of 43.0±13.8 years, 83.5% men, HBV co-infection percentage of 4%, HCV co-infection percentage of 2.5%, HIV viral load of mean 4.7±0.7 log copies/ml and CD4 cell counts of mean 109±74 cells/μl (Table 1). PP analysis showed 67.9% (57/84) of patients in group B had achieved viral suppression (< 50 copies/mL) at 12 weeks in comparison to 48.1 % (37/77) of that in group A (p< 0.01). The mean viral load levels decreased sharply in the drug B group from 4.70 ± 0.72 to 1.62 ± 0.47 log copies/ml over 12 weeks, compared to group A (p< 0.01, figure 1). However, in the two groups, CD4 counts had a similar change trend over 24 weeks and the proportion of CD4 cell counts over 200 cells/μl remained identical (figure 2). ITT analysis further demonstrated the results above.

**Figure:**
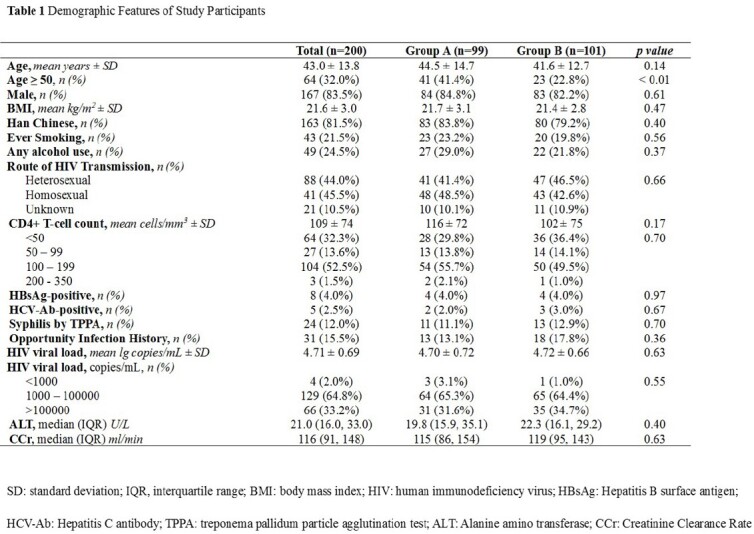

**Figure:**
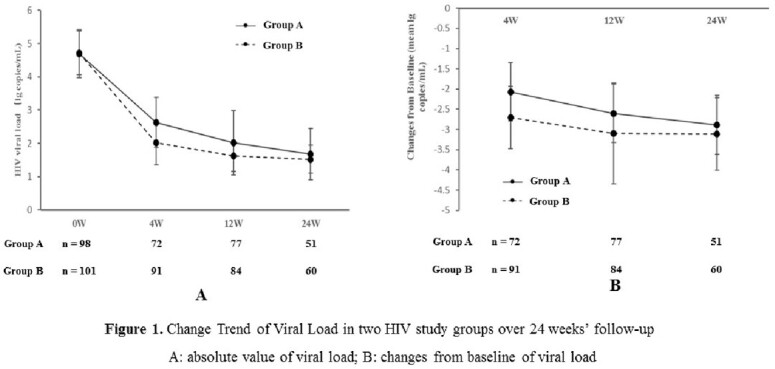

**Figure:**
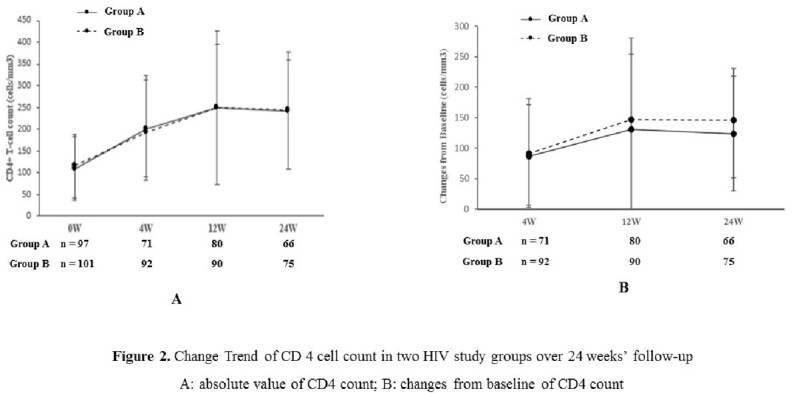

**Conclusion:**

Compared to TDF/3TC/EFV, BIC/FTC/ TAF as the initial choice for late presenters had better performance to achieve rapid viral suppression, especially in the first three months therapy, but showed no superiority in immune deficiency improvement.

**Disclosures:**

**All Authors**: No reported disclosures

